# T2 relaxation time for the early prediction of treatment response to chemoradiation in locally advanced rectal cancer

**DOI:** 10.1186/s13244-022-01254-z

**Published:** 2022-07-07

**Authors:** Yuxi Ge, Yanlong Jia, Xiaohong Li, Weiqiang Dou, Zhong Chen, Gen Yan

**Affiliations:** 1 Department of Radiology, Affiliated Hospital of Jiangnan University, Wuxi, Jiangsu China; 2 Department of Radiology, Xiangyang Central Hospital, Affiliated Hospital of Hubei University of Arts and Science, Xiangyang, Hubei China; 3 GE Healthcare, MR Research China, Beijing, China; 4 School of Electronic Science and Engineering, Xiamen University, Xiamen, Fujian China; 5 Department of Radiology, The Second Affiliated Hospital of Xiamen University, Xiamen, 361021 Fujian China

**Keywords:** Chemoradiotherapy, T2 relaxation time, DWI, Rectal cancer, Response

## Abstract

**Objectives:**

Poor responders to chemoradiotherapy (CRT) for locally advanced rectal cancer (LARC) can still have a good prognosis if the treatment strategy is changed in time. However, no reliable predictor of early-treatment response has been identified. The purpose of this study was to investigate the role of T2 relaxation time in magnetic resonance imaging (MRI) for the early prediction of a pathological response to CRT in LARC.

**Methods:**

A total of 123 MRIs were performed on 41 LARC patients immediately before, during, and after CRT. The corresponding tumor volume, T2 relaxation time, and apparent diffusion coefficient (ADC) values at different scan time points were obtained. The Mann–Whitney *U* test was used to compare the T2 relaxation time between pathological good responders (GR) and non-good responders (non-GR). The area under the curve (AUC) value was used to quantify the diagnostic ability of each parameter in predicting tumor response to CRT.

**Results:**

Twenty-one (51%) and 20 (49%) were GRs and non-GRs, respectively. T2 relaxation time showed an excellent intraclass correlation coefficient (ICC) of > 0.85 at three-time points. It was significantly lower in the GR group than in the non-GR group during and after CRT. The early T2 decrease had a high AUC of 0.91 in differentiating non-GRs and GRs, similar to 0.90 of the T2 value after CRT.

**Conclusions:**

T2 relaxation time may help predict treatment response to CRT for LARC earlier, rather than having to wait until the end of CRT, thereby alleviating the physical burden for patients with no good response.

**Supplementary Information:**

The online version contains supplementary material available at 10.1186/s13244-022-01254-z.

## Key points

1. T2 relaxation time in rectal cancer has high inter-observer agreement.
2. The early T2 decrease is higher in good responders than non-good responders.
3. The early T2 decrease enables excellent diagnostic accuracy in predicting CRT response.

## Background

Preoperative chemoradiotherapy (CRT) followed by total mesorectal excision (TME) is the standard treatment modality for locally advanced rectal cancer (LARC) [1]. The outcomes of CRT are a crucial endpoint in LARC management, with 14–36% achieving a pathological complete response (pCR) [2, 3]. A wait-and-watch policy [4, 5] has been proposed for these patients because it may have similar disease-free survival and overall survival benefit to surgery. However, a small proportion of patients fail to benefit from CRT [6] and develop toxicity and tumor progression during treatment. These poor responders may obtain an improved prognosis if the treatment strategy changes in time to begin intensive chemotherapy, immunotherapy [7], or heavy particle therapy [8]. Thus, early prediction of the treatment outcomes is crucial. However, no reliable predictor for such purpose has been identified; thus, there is an urgent need for reliable imaging markers to distinguish responders from non-responders in the early stages of CRT.

Several studies have attempted to use magnetic resonance imaging (MRI) to predict early CRT response in LARC. Tumor size reduction on early MRI during CRT for LARCs can distinguish complete response (CR), partial response (PR), and non-response (NR) with high accuracy [9]. A clinical trial also supported the predictive value of tumor volume regression during CRT [10]. In addition to reducing tumor volumetry, functional changes have also been detected in the early stages of CRT. Diffusion-weighted imaging (DWI) is the most commonly used method for evaluating the CRT response. It has been proven useful for selecting good treatment responders during preoperative CRT for LARCs [11, 12]. However, conventional DWI sequences have essential limitations and presence of artifacts that lead to a lower resolution than high-resolution T2-weighted imaging (T2WI), the basic sequence for preoperative evaluation of rectal cancer. In recent years, texture and radiomics features based on T2WI are increasingly applied to predict pCR in LARC due to their better classification performance [13, 14], ultimately proving the importance of T2WI in MRI.

T2-weighted images demonstrate the tissue's relative, not absolute, T2 signal intensity. T2 mapping calculates each tissue's intrinsic T2 relaxation time by fitting an exponential function to the signal intensity at different echo times. T2 relaxation time is an absolute quantitative value with high reproducibility [15] that may have a higher potential for assessing the response to CRT in LARC. This study aimed to investigate the feasibility and reproducibility of T2 mapping during CRT compared to DWI for the early prediction of histopathological tumor regression (TRG). We hypothesized that T2 relaxation time would be identified in the early phases of CRT for LARC.

## Materials and methods

### Study design and patients

This prospective study was approved by the Medical Ethics Committee of our institution and conformed to the tenets of the Declaration of Helsinki. Written informed consent was obtained from all patients.

**Fig. 1:**
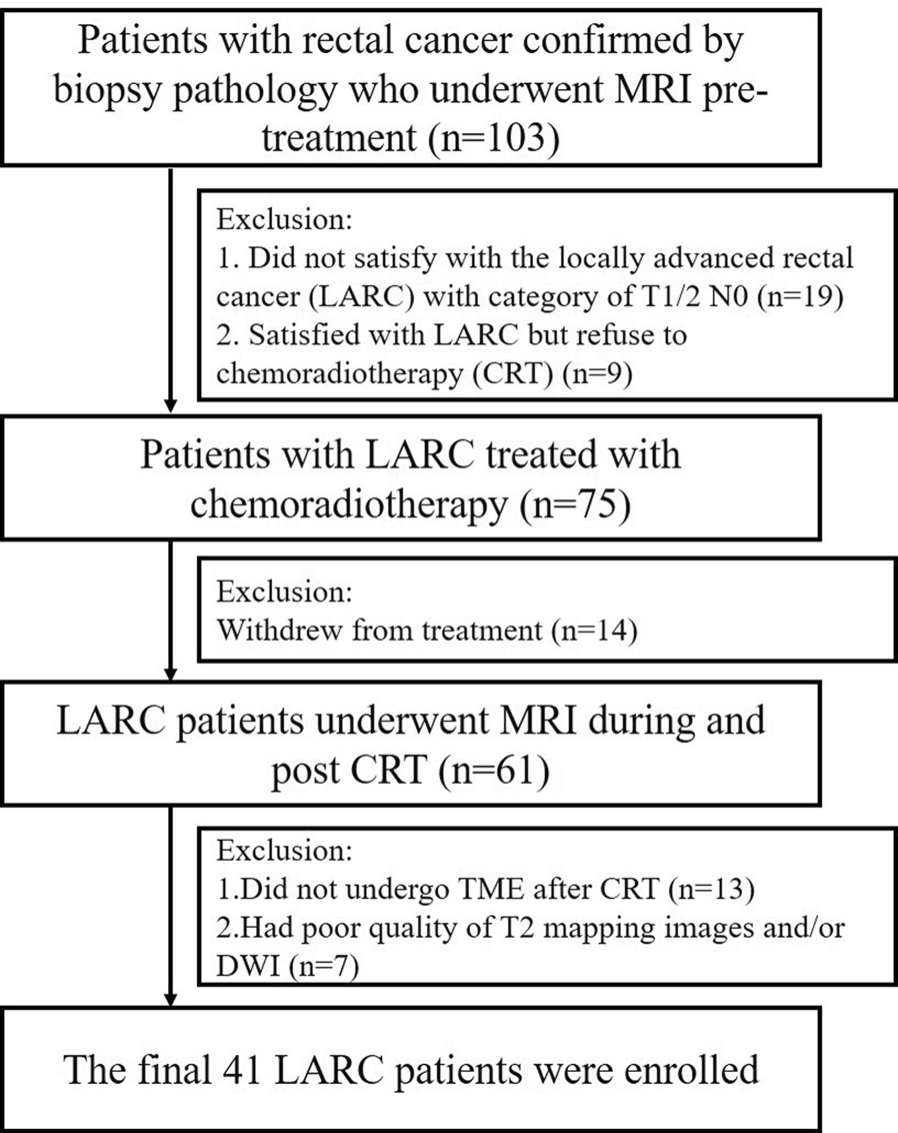
Flowchart shows patient selection process based on inclusion and exclusion criteria

One hundred and three patients diagnosed pathologically with rectal adenocarcinoma between January 2018 and April 2021 were enrolled. Patients who (a) did not satisfy the LARC diagnostic criteria on primary staging MRI scan (category cT1-2 and node-negative status, *n* = 19); (b) refused preoperative chemoradiation or had received chemoradiation, but withdrew from treatment (*n* = 23); (c) did not undergo TME after CRT (*n* = 13); and (d) had poor quality of T2 mapping images, diffusion-weighted images, or both (*n* = 7), were excluded. Finally, 41 adult patients with pathologically proven rectal adenocarcinomas who underwent preoperative CRT and surgical resection were evaluated (Fig. 1). MRI, including T2WI, DWI, and T2 mapping, was performed before, during, and after CRT.

### Treatment

Preoperative CRT included initial induction chemotherapy, subsequent CRT, and consolidation chemotherapy according to the National Comprehensive Cancer Network (NCCN) guidelines. Compared with short-course radiotherapy, a long-course radiotherapy was administered in our institution because it is a more frequently chosen neoadjuvant CRT plan in China with fewer adverse events. The patients received a dose cycle of oxaliplatin (130 mg/m^2^, day 1) plus capecitabine (825 mg/m^2^, days 1–14), also known as CapeOx, once every 3 weeks before radiotherapy.

The subsequent radiotherapy was administered once a day at 1.8–2.0 Gy/fraction per day, 5 days per week, for a total dose of 50–50.4 Gy in 25–28 fractions. A CT scan was acquired at 3 mm slice thickness for planning purpose. Most patients were immobilized in the prone position with an ankle-holder. The radiation clinical target volume (CTV) included the primary rectal cancer, perirectal and internal iliac nodes, mesorectum, pelvic sidewalls, and presacral space with the upper border at the sacral promontory. Intensity-modulated radiotherapy (IMRT) treatment with 6–10-MV X-rays delivered using a Varian linear accelerator was administered with daily image guidance. Oral capecitabine (825 mg/m^2^, bis in die [b.i.d.]) was administered during the radiotherapy sessions.

Consolidation chemotherapy was conducted approximately 2 weeks after the completion of CRT. Consolidation chemotherapy involved two or three cycles of CapeOx, with the same dose as induction chemotherapy. TME was performed 8–11 weeks after radiation therapy.

### Magnetic resonance imaging

The MRI assessment consisted of one MRI scan during CRT at the end of radiation therapy (8 weeks after the start of the treatment), in addition to the standard-of-care MRI scans, pre-CRT (at the beginning of treatment), and post-CRT (8 weeks after the end of radiation therapy). A 3.0 Tesla magnetic resonance (MR) system (750 w; GE, Milwaukee, WI, USA) with a 16-channel phased-array body coil was used for image acquisition. All the patients were scanned in the supine position with a feet-first orientation. Rectal preparation involved fasting and taking oral polyethylene glycol electrolyte solution to empty the intestinal contents 6–8 h before the examination. Polyethylene glycol electrolyte was used because, as we observed in the preliminary experiments, it can discharge not only waste but also the air in the rectum, thus producing better T2WI tissue contrast and reducing distortions in DWI (Additional file 1: Fig. S1). Unless contraindicated, 20 mg of raceanisodamine hydrochloride (Minsheng Pharmaceutical Group Co., Ltd., Hangzhou, China) was slowly injected intramuscularly into the buttocks to prevent bowel peristalsis approximately 10–15 min before the examination.

Axial, sagittal, and coronal high-resolution T2-weighted (3227–5326 ms/102 ms, that is repetition time ms/echo time ms]; field of view, 24 cm × 24 cm to 30 cm × 30 cm; flip angle, 111°; matrix size, 320 × 224; 3 mm/0.3 mm [slice thickness/spacing]; number of sections, 18–28; acquisition time, 2 min 24 s to 2 min 35 s) and axial echo-planar diffusion-weighted images (3141 ms/71 ms; field of view, 36 cm × 36 cm; matrix size, 128 × 128; 5 mm/1 mm; *b*-value, 0 and 1000 s/mm^2^; number of sections, 18; acquisition time, 2 min 3 s) were obtained. The oblique axial planes were orthogonal to the tumor, based on the high-resolution T2W images. Coronal T2 mapping (1500 ms/8.3 ms, 16.4 ms, 24.5 ms, 32.6 ms, 40.7 ms, 48.8 ms, 56.9 ms, 65 ms; field of view, 30 cm × 30 cm; flip angle, 142°; bandwidth, 31.25 Hz/pixel; matrix size, 256 × 160; 3 mm/0.3 mm; number of sections, 18; acquisition time, 4 min 30 s) was performed to cover all tumor layers in the shortest possible time with a location line parallel to the long axis of the tumor.

### Image measurements

DWI and T2 maps were transferred to a workstation (ADW 4.6, workplace; General Electric). A senior radiologist and a junior radiologist (X.L. and Y.J., with 25 and 6 years of gastrointestinal tumor diagnostic experience, respectively) who were blinded to the study design and diagnoses performed the measurements. The junior radiologist repeated the measurements 2 weeks later. To determine the ADC, the tumor's region of interest (ROI) was drawn freehand on high *b*-value DWIs (*b* = 1000 s/mm^2^) along the tumor border on each tumor slice about the corresponding T2W image. For the T2 relaxation time, the ROI was drawn freehand on the original T2 mapping image of 65 ms echo time, along the border of the tumor, on each slice.

The relative early tumor volume decrease (Δ*V*_during_) was calculated using the *V*_pre_ and *V*_during_ (*V*_during_ − *V*_pre_) × 100%/*V*_pre_. The relative early ADC_increase_ (ΔADC_during_) was calculated using the ADC_pre_ and ADC_during_ (ADC_during_ − ADC_pre_) × 100%/ADC_pre_. The relative early T2 decrease (ΔT2_during_) was calculated using the following equation: ΔT2_during_ = (T2_pre_ − T2_during_) × 100%/T2_pre_.

### Pathological analysis

Histopathological examinations were performed according to the criteria of the American Joint Committee on Cancer (AJCC) TNM Classification of Malignant Tumors 8th edition. All the examinations were performed using resection specimens obtained by different experienced pathologists and reviewed by a single dedicated gastrointestinal pathologist who was blinded to the MRI data. The pathological tumor regression grade (TRG) was classified as follows, according to Mandard et al. [16]: TRG 1, pathological complete response (pCR); TRG 2, rare residual tumor cells; TRG 3, more residual tumor cells with the preponderance of fibrosis; TRG 4, residual tumor cells outgrowing fibrosis; and TRG 5, absence of regressive changes. The good response (GR) group involved patients with Mandard TRG 1–2, while the non-good response (non-GR) group involved those with a moderate or poor response (TRG 3–5).

### Statistical analysis

Intra- and inter-observer correlations for the T2 and ADC values were analyzed by estimating the ICCs. The values obtained by the radiologists were used for further statistical testing only when the intra- and inter-observer ICC values were greater than 0.75, which indicated excellent consistency. The histopathological correlations were analyzed using Spearman's correlation test. The Mann–Whitney *U* test was used for ADC values, T2 relaxation time, tumor volume, and the corresponding ratios. The area under the receiving operating characteristic curves (AUC) was used to compare the diagnostic power of these test indicators. The cutoff values were determined, and their sensitivity and specificity were calculated. De Long's test was used to compare the difference between AUCs. The differences were considered statistically significant if the *p*-value was less than 0.05. The statistical analyses were performed using the Statistical Package for the Social Sciences (SPSS version 22.0; IBM Corp., Armonk, NY) and MedCalc Statistical Software (MedCalc Software Ltd, version 18.2, Ostend, Belgium).

## Results

### Patient characteristics

In total, 41 patients (23 men, 18 women) with a mean age of 64 y (31–81 y) were included in the study. All the patients completed the long-course CRT and TME. After the histopathological examination, 21 patients (51%) were classified into the GR group; among them, six achieved pCR. Meanwhile, the remaining 19 patients (46%) were classified into the non-GR group. The patient characteristics are summarized in Table 1.

**Table 1:** Patient characteristics

Characteristic	Value
*Sex, n (%)*	
Male	23 (56)
Female	18 (44)
*Age (years), median (range)*	64 (31–81)
*Tumor location, n (%)*	
Distal rectum	13 (32)
Middle	17 (41)
Proximal rectum	11 (27)
*Clinical T classification, n (%)*	
T3	23 (56)
T4	18 (44)
*Clinical N classification, n (%)*	
N0	4 (10)
N+	37 (90)
*ypT classification, n (%)*	
0	6 (15)
1	13 (38)
2	12 (29)
3	5 (12)
4	5 (12)
*ypN classification, n (%)*	
N0	22 (54)
N +	19 (46)
*Pathological TRG, n (%)*	
1	6 (15)
2	16 (39)
3	8 (20)
4	7 (17)
5	4 (10)
*Operative procedure, n (%)*	
Low anterior resection	27 (66)
Ultra-low anterior resection	5 (12)
Intersphinteric resection	4 (10)
Abdominoperineal resection	5 (12)

### Intra- and inter-observer agreement

The T2 relaxation time displayed excellent intra- and inter-observer agreement before, during, and after CRT, with ICCs of 0.95, 0.92; 0.95, 0.95; and 0.94, 0.82, respectively. Comparable results were also found for the tumor volumes and ADC values. However, the ADC values after CRT showed the lowest ICCs, 0.82 and 0.64 for intra- and inter-observer agreements, respectively. Therefore, the ADC values after CRT were not used for further analyses. The details of ICCs are shown in Table 2. The first measurements obtained by the junior radiologist were used for further analyses.

**Table 2:** Consistency evaluation for tumor volume, ADC and T2 relaxation time at different time points

ICC	Tumor volume		ADC value		T2 value	
	Intra-observer	Inter-observer	Intra-observer	Inter-observer	Intra-observer	Inter-observer
Before CRT	0.99 (0.99–1.00)	0.99 (0.99–1.00)	0.98 (0.96–0.99)	0.96 (0.92–0.98)	0.95 (0.85–0.98)	0.92 (0.86–0.96)
During CRT	0.99 (0.96–1.00)	0.98 (0.97–0.99)	0.95 (0.90–0.97)	0.91 (0.83–0.95)	0.95 (0.90–0.97)	0.95 (0.88–0.98)
After CRT	0.99 (0.99–1.00)	0.99 (0.99–1.00)	0.82 (0.67–0.90)	0.64 (0.33–0.80)	0.94 (0.86–0.97)	0.82 (0.33–0.93)

CRT, chemoradiotherapy; ICC, intra-class correlation coefficient

### Correlation with pathological TRG

T2_during_, T2_post_, and ΔT2_during_ showed significant correlation with the pathologic TRG, with high correlation coefficients (*r* = 0.586, 0.867, and − 0.753, respectively). The *V*_post_, Δ*V*_during_, and ΔADC_during_ also demonstrated a moderate correlation coefficient (*r* = 0.404, − 0.485, and 0.485, respectively). However, neither the tumor volume nor ADC values pre and during CRT correlated with pathology. The correlation coefficients are listed in Table 3.

**Table 3:** Comparison of T2 and ADC values between good responders and non-good responders and the correlation test

	TRG 1–2 (median, interquartile distance)	TRG 3–5 (median, interquartile distance)	Mann–Whitney *U* (Z, *P*)	Rank correlation with TRG (*r*, *P*)
*V*_pre_ (cc)	18.3 (11.2)	16.3 (10.9)	− 0.765, 0.445	− 0.202, 0.206
*V*_during_ (cc)	10.0 (5.7)	10.2 (6.5)	0.652, 0.514	− 0.003, 0.983
*V*_post_ (cc)	6.0 (2.8)	7.8 (6.0)	3.134, 0.002*	0.404, 0.008*
Δ*V*_during_ (%)	45.9 (15.6)	26.8 (21.0)	3.234. 0.001*	− 0.485, 0.001*
ADC_pre_ (× 10^−3^ mm^2^/s)	1.21 (0.71)	1.24 (0.65)	− 1.601, 0.109	0.283, 0.073
ADC_during_ (× 10^−3^ mm^2^/s)	1.34 (0.21)	1.36 (0.56)	− 0.384, 0.701	0.026, 0.873
ΔADC_during_ (%)	25 (32)	10 (19)	− 2.914, 0.004*	0.485, 0.001*
T2_pre_ (ms)	71.0 (5.0)	68.5 (13.0)	− 0.929, 0.350	− 0.108, 0.500
T2_during_ (ms)	61.0 (5.0)	65.5 (9.0)	− 2.800, 0.005*	0.586, < 0.001*
T2_post_ (ms)	53.0 (6.0)	61.0 (8.0)	4.390, < 0.001*	0.857, < 0.001*
ΔT2_during_ (%)	13.9 (9.3)	4.5 (8.5)	− 4.474, < 0.001*	− 0.753, < 0.001*

*p < 0.05

*V*_pre_: tumor volume at pre-treatment MRI; *V*_during_: tumor volume during CRT; *V*_post_: tumor volume after CRT; Δ*V*_during_: the relative early tumor volume decreased from beginning to the middle of CRT; ADC_pre_: ADC values at pre-treatment MRI; ADC_during_: ADC values of MRI during CRT; ΔADC_during_: the relative early ADC increase from beginning to middle of CRT; T2_pre_: T2 relaxation time at pre-treatment MRI; T2_during_: T2 relaxation time of MRI during CRT; T2_post_: T2 relaxation time of MRI after CRT; ΔT2_during_: the relative T2 increase from initiation to the end of radiotherapy

Cc, cubic centimeter; ms, millisecond

### T2 relaxation time between GRs and non-GRs

**Fig. 2:**
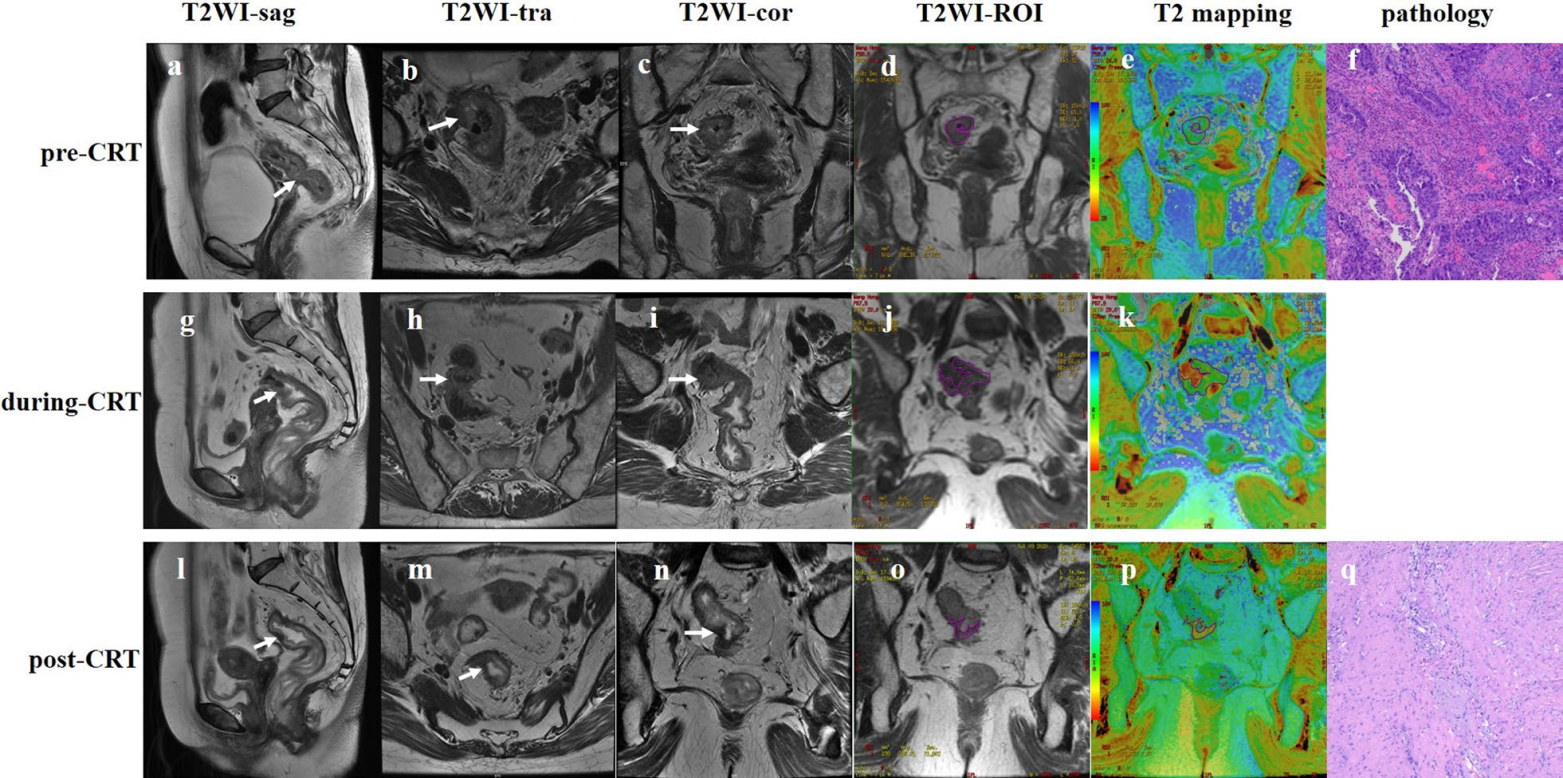
MR images of a 52-year-old woman with good response to chemoradiotherapy. Baseline sagittal (**a**), axial (**b**), and coronal (**c**) T2-weighted images showing neoplastic tissue in the upper rectum with diffuse infiltration of the rectal wall and mesorectal fat (T3). The freehand ROI is drawn on the original image of the T2 maps (**d**), and then 72 ms of T2 value is calculated accordingly, which appears green on the color-coded image (**e**). This patient is confirmed to have tubular adenocarcinoma by biopsy (**f**, hematoxylin–eosin stain, original magnification ×100). At the end of radiotherapy, the tumor volume is reduced (**g**–**i**), the T2 value of the tumor is decreased to 60 ms (**j**, **k**), and the relative T2 is decreased to 16.7%. After CRT, the tumor volume is further reduced (**l**–**n**), and the T2 value is decreased to 52 ms (**o**, **p**) (the patchy red areas on the color-coded image represent fibrosis). The postoperative pathology is classified as TRG 1, with no residual tumor cells found in fibrosis (**q**, hematoxylin–eosin stain, original magnification ×100)

**Fig. 3:**
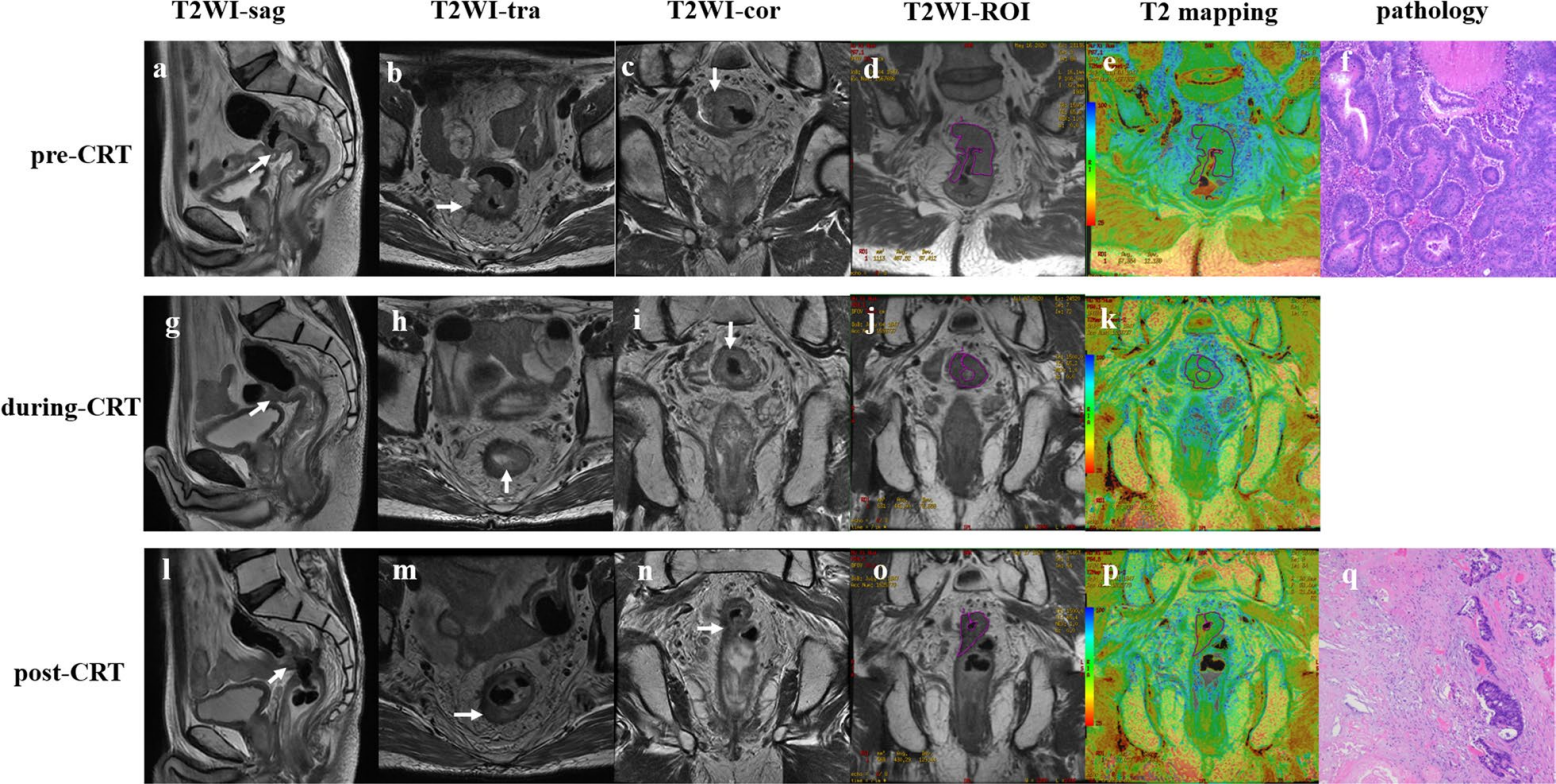
MR images of a 74-year-old man with poor response to chemoradiotherapy. Baseline sagittal (**a**), axial (**b**), and coronal (**c**) T2-weighted images showing neoplastic tissue in the upper rectum with diffuse infiltration to the rectal wall and mesorectal fat (T3). The freehand ROI is drawn on the original image of the T2 maps (**d**), and then the 67 ms T2 value is calculated accordingly, which appears green on the color-coded image (**e**). This patient is confirmed to have tubular adenocarcinoma on biopsy (**f**, hematoxylin–eosin stain, original magnification ×100). At the end of radiotherapy, the tumor volume is reduced (**g**–**i**), and the T2 value decreased to 62 ms (**j**, **k**), and the relative T2 decreased to 7.4%. The tumor volume was further reduced after CRT (**l**–**n**), and the T2 value decreased to 57 ms (**o**, **p**) (the dotted red areas on the color-coded image represent the components of interstitial fibrosis). The postoperative pathology is TRG 3, with more residual tumor cells in fibrosis (**q**, hematoxylin–eosin stain, original magnification ×100)

The GR group (Fig. 2) showed significantly lower T2 relaxation time during (61 ms vs. 65.5 ms, *p* = 0.003) and post (53 ms vs. 61 ms, *p* < 0.001) CRT than the non-GR group (Fig. 3). Comparable results were found for ΔT2_during_ (13.9% vs. 4.5%, *p* < 0.001), ΔADC_during_ (29.5% vs. 6.2%, *P* = 0.001), and Δ*V*_during_ (45.9% vs. 26.8%, *P* = 0.001). Meanwhile, there were no significant between-group differences in the ADC values and tumor volumes before and during CRT (Table 3). The T2 and ADC analyses are summarized in Table 3.

### Predictive values

**Fig. 4:**
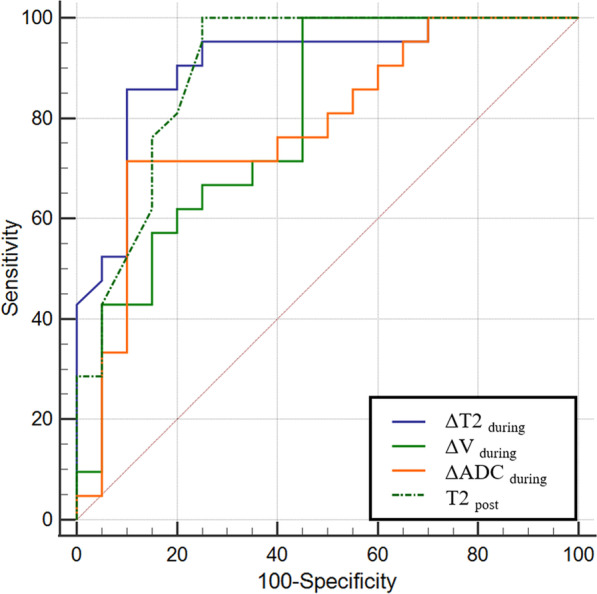
Receiver operating characteristics (ROC) curve of the early T2decrease, early volume decrease, early ADC values increase, and post-CRT T2 values for predicting the response to CRT in rectal cancer

Sensitivity, specificity, and accuracy in predicting pathological non-good response patients for ΔT2_during_ were 90%, 86% and 88%, and 55%, 100%, 76% for Δ*V*_during_, and 90%, 67%, 78% for ΔADC_during_, respectively. T2_post_ showed lower sensitivity (75%) and higher specificity (100%) compared to ΔT2_during_ the procedure. Although ΔT2_during_ achieved a higher AUC of 0.91, Delong's test showed no statistical difference between ΔT2_during_ and Δ*V*_during_, ΔADC_during_, and post-T2 values (*p* values = 0.160, 0.153, 0.872, respectively) (Table 4 and Fig. 4).

**Table 4:** Comparison of predictive performance for non-good pathological response between tumor volume, T2 and ADC values

	Sensitivity (%)	Specificity (%)	Accuracy (%)	Cutoff value	AUC (95% CI)
Δ*V*_during_	55 (11/20)	100 (21/21)	76 (31/41)	28%	0.79 (0.64–0.91)
ΔADC_during_	90 (18/20)	67 (14/21)	78 (32/41)	19%	0.78 (0.63–0.90)
ΔT2_during_	90 (18/20)	86 (18/21)	88 (36/41)	11%	0.91 (0.78–0.98)
T2_post_	75 (15/20)	100 (21/21)	86 (36/41)	58 ms	0.90 (0.77–0.97)

ΔADC_during_: the relative early ADC increase from beginning to the end of radiotherapy; T2_during_: T2 relaxation time of MRI during CRT; T2_post_: T2 relaxation time of MRI post CRT; ΔT2_during_: the relative early T2 decrease from initiation to the end of radiotherapy

## Discussion

Reliable and quantitative MRI values for the early monitoring of treatment responses are critical for individualized treatment decisions; however, no imaging markers have been established to distinguish the responders from non-responders in the early stages of CRT. This study found that T2 relaxation time is a potentially valuable indicator of treatment response to CRT for LARC, as shown by their excellent ICC values and high correlation coefficients with the pathologic TRG. The relative early T2 decrease during CRT was significantly higher in the GR group than in the non-GR group. It showed a diagnostic efficacy similar to the T2 relaxation time at the end of CRT. To the best of our knowledge, this study was the first to investigate the feasibility and reproducibility of T2 relaxation time for predicting a pathological response during CRT in patients with LARC.

T2 mapping is considered a robust technology for myocardial [15, 17] and prostatic [18] T2 value measurements. In our study, T2 relaxation time showed high intra- and inter-observer consistency in rectal tumor measurements before, during, and after CRT. In the gastrointestinal tract, the high consistency of T2 value measurements for lymph nodes in rectal cancer was confirmed [19, 20]. However, the ADC values are less reproducible for the measurement of rectal cancer after CRT because the tumor ADC values are highly dependent on the ROI positioning method used [21]. In the current study, ADC after CRT for rectal cancer had moderate reproducibility. In contrast, ADC before and during CRT had excellent reproducibility, as agreed on by a previous report of a 0.66 ICC for post-CRT ADC values [22]. Due to their low resolution, it is difficult to identify the tumor boundaries and microstructures in DWI and ADC maps. In contrast, the tumor boundaries can be clearly distinguished on T2 maps due to the high resolution.

Several approaches have been attempted for the early evaluation of tumor response using quantitative MRI parameters, including tumor shrinkage-based T2WI [10, 23], radiomics based on pre and early-treatment MRI [24], and the ADC values obtained mid-MRI [12, 14]. In line with previous data, we found that relatively increased ADC and decreased tumor volume during CRT were potential predictors of postoperative pathological response to CRT, with a moderate correlation coefficient with the pathological TRG. Compared with relatively increased ADC and decreased tumor volume during CRT, the relative T2 decrease offered may provide more helpful information about the tumor microstructural changes, such as fibrous and residual tumor changes due to the higher correlation coefficient with the pathological TRG. The T2 relaxation time indicated the tissue's water content, especially free water molecules; thus, they decrease in a fibrotic myocardium [25, 26] and increase in an edematous myocardium [27].

Different collagen fibers may appear during CRT in the tumor, especially in GRs [28]. This development reduces the water content of the tumor and results in a more noticeable decrease in the T2 value of the GRs. The decline in T2 relaxation time was first identified in patients with breast cancer after neoadjuvant chemotherapy [29]. Similarly, in rectal cancer patients, T2 relaxation time had varying degrees of decrease during the CRT, while those with less residual tumor had a higher T2 decreased rate. It was reported that the split scar with hypointensity on T2-weighted image was characteristic of CRs, while intermediate signals may represent residual tumor after CRT for LARC [30]. Consistent with the morphologic patterns visible on T2-weighted images, we found a high correlation between T2 relaxation time and the proportion of residual tumor cells and fibrous components in the tumor after CRT.

Some prospective exploratory studies reported that MRI during neoadjuvant RCT may predict early pCR in rectal cancer patients [31]. A radiomics model that included MRI values before and during radiotherapy showed excellent predictive capability for identifying pCR in LARC patients, with an AUC of 0.93 [24]. Early reduction rates in the tumor volume (ΔV_T2WI_) showed an AUC of 0.899 for predicting CR after CRT [23]. Our study's early T2 decrease had an AUC of 0.91 for predicting tumor response. T2 relaxation times offer a simpler quantitative measurement, which can be implemented at the MRI workstation, as they are strictly based on the visual morphology of T2WI. Our analyses before, during, and after CRT for LARC showed that T2 relaxation time has high reproducibility. Further, it eliminates the need for laborious image segmentation in radiomics analysis.

Our study had some limitations. First, the number of patients included in the present study was limited. More prospective studies with larger numbers of patients are needed to validate our results. Therefore, the proposed T2 cutoff values should be considered cautiously and need to be verified in larger, multicenter studies and other MRI scans. Second, using basic T2 mapping to evaluate the entire tumor area was time-consuming. However, accelerated T2 mapping may be more time-efficient than conventional T2 mapping in the prostate [32]. Third, our data acquisition may have been biased because the T2 relaxation time was measured in the coronal view while the ADC values were measured in the axial view. Fourth, polyethylene glycol electrolyte was used before the MRI examination in this study. Although it played a key role, larger samples and multicenter samples were needed to confirm its reliability. Finally, more quantitative multiparametric MRIs may also offer potential imaging markers, such as T1, PD, and T2* values, for the early prediction of treatment response to neoadjuvant therapy.

## Conclusions

For the first time, this study investigated the clinical value of T2 mapping in predicting treatment response to CRT for LARC patients. Significantly different T2 values were found between GR and non-GR groups, and a high correlation relationship was revealed between T2 and pathologic TRG. Additionally, ΔT2_during_ was significantly higher in the GR group than in the non-GR group and provided a robust diagnostic efficacy in distinguishing non-GRs from GRs. This finding indicates that ΔT2_during_, calculated using T2 before and during CRT, can evaluate the CRT response early, rather than waiting until the end of CRT, alleviating the physical burden for patients with no good response.

## Supplementary Information

**Additional file 1: Fig. S1**. Figures** a**,** b**,** c** were MR images of a patient with oral polyethylene glycol electrolyte solution. On sagittal T2 weighted images (**a**), the tumor contour was clearly shown against the water in the intestinal lumen. A region of interest (ROI) was outlined along the contour of the intestinal lumen at axial T2WI (shown as the red solid line,** b**). Then, this ROI was copied onto the DWI (shown as the red dashed line). Subsequently, another ROI was outlined on DWI using the same method (shown as the green dashed line). The two ROIs were well matched (**c**). Figures** d**,** e**,** f** were MR images of a patient without any bowel preparation. The tumor contour was difficult to be distinguished from the surrounding tissues due to the influence of faeces and air in the intestinal lumen on sagittal T2WI (**d**). A ROI was outlined along the contour of the intestinal lumen at axial T2WI (shown as the red solid line,** e**). Then, this ROI was copied onto the DWI (shown as the red dashed line). Subsequently, another ROI was outlined on DWI along the contour of the intestinal lumen (shown as the green dashed line). The two ROIs cannot be well matched with large morphological variation, suggesting significant DWI distortion. t: tumor; w: water; f: faeces.

## Data Availability

The datasets used and/or analyzed during the current study are available from the corresponding author on reasonable request.

## Abbreviations

ADC: Apparent diffusion coefficient
CRT: Chemoradiotherapy
GR: Good responders
ICC: Intraclass correlation
LARC: Locally advanced rectal cancer
ROI: Region of interest
TRG: Tumor regression grade
