# Supplementary material for: T2 relaxation time for the early prediction of treatment response to chemoradiation in locally advanced rectal cancer
Source: Insights Imaging. 2022 Jul 7;13:113. doi: 10.1186/s13244-022-01254-z (PMC9263013; doi:10.1186/s13244-022-01254-z)

ELECTRONIC SUPPLEMENTARY MATERIAL

T2 relaxation time for the early prediction of treatment response to chemoradiation in locally advanced rectal cancer

Supplementary Figure 1

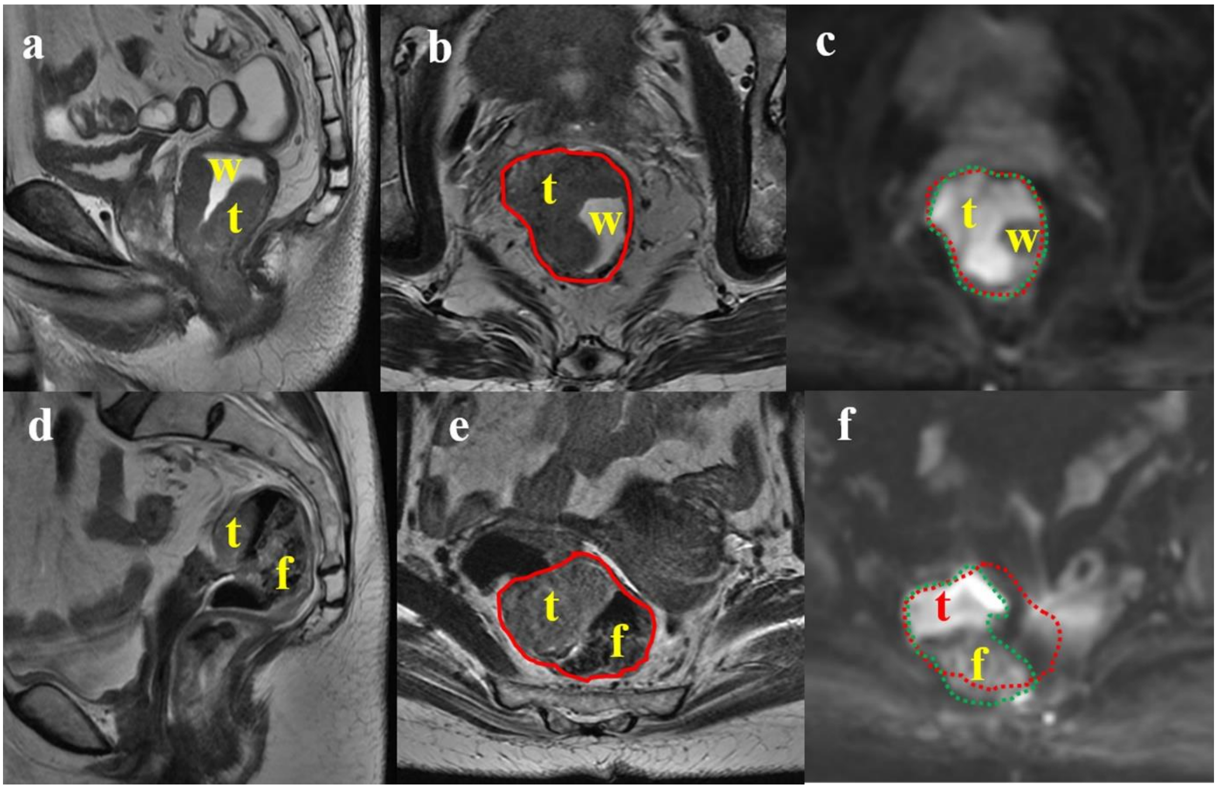

Supplement: Supplementary file 1 — Additional file 1: Fig. S1. Figures a, b, c were MR images of a patient with oral polyethylene glycol electrolyte solution. On sagittal T2 weighted images (a), the tumor contour was clearly shown against the water in the intestinal lumen. A region of interest (ROI) was outlined along the contour of the intestinal lumen at axial T2WI (shown as the red solid line, b). Then, this ROI was copied onto the DWI (shown as the red dashed line). Subsequently, another ROI was outlined on DWI using the same method (shown as the green dashed line). The two ROIs were well matched (c). Figures d, e, f were MR images of a patient without any bowel preparation. The tumor contour was difficult to be distinguished from the surrounding tissues due to the influence of faeces and air in the intestinal lumen on sagittal T2WI (d). A ROI was outlined along the contour of the intestinal lumen at axial T2WI (shown as the red solid line, e). Then, this ROI was copied onto the DWI (shown as the red dashed line). Subsequently, another ROI was outlined on DWI along the contour of the intestinal lumen (shown as the green dashed line). The two ROIs cannot be well matched with large morphological variation, suggesting significant DWI distortion. t: tumor; w: water; f: faeces. [file 13244_2022_1254_MOESM1_ESM.pdf]
